# An improved method to prepare an injectable microemulsion of the galanin-receptor 3 selective antagonist, SNAP 37889, using Kolliphor^®^ HS 15

**DOI:** 10.1016/j.mex.2014.09.003

**Published:** 2014-09-26

**Authors:** Karlene J. Scheller, Spencer J. Williams, Andrew J. Lawrence, Bevyn Jarrott, Elvan Djouma

**Affiliations:** a Department of Human Biosciences, La Trobe University, Bundoora, Victoria, Australia; b School of Chemistry and Bio21 Molecular Science and Biotechnology Institute, University of Melbourne, Parkville, Victoria, Australia; c Florey Institute of Neuroscience & Mental Health, University of Melbourne, Parkville, Victoria, Australia; d Melbourne Brain Centre, University of Melbourne, Parkville, Victoria, Australia

**Keywords:** Injectable microemulsion of SNAP 37889, Galanin-3 receptor, Galanin-3 antagonist, SNAP 37889, Kolliphor^®^ HS 15, Solubility, i.p. injection, Drug delivery, Microemulsion

## Abstract

Research into the galanin-3 (GAL3) receptor has many challenges, including the lack of commercially available selective ligands. While the identification of non-peptidergic GAL3 receptor-selective antagonists, 1-phenyl-3-[3-(trifluoromethyl)phenyl]iminoindol-2-one (SNAP 37889) and 1-[3-(2-pyrrolidin-1-ylethoxy)phenyl]-3-[3-(trifluoromethyl)phenyl]iminoindol-2-one (SNAP 398299) have implicated a role for GAL3 receptors in anxiety, depression and drug-seeking behaviour, a major limitation of their use is poor aqueous solubility. Previously we have used 5% dimethylsulfoxide (DMSO) with 1% hydroxypropylmethyl cellulose in saline to dissolve SNAP 37889 for intraperitoneal (i.p.) injections of rats; however this produced a micro-suspension that was not ideal. The injectable formulation of SNAP 37889 was improved as follows:

• 30% (w/v) Kolliphor^®^ HS 15 (Solutol HS^®^ 15) and sodium phosphate buffer (0.01 M, pH 7.4) were used as vehicles.
• A smooth glass mortar and pestle was used to triturate the Kolliphor^®^ HS 15 and SNAP 37889 into a paste before addition to the sodium phosphate buffer at room temperature (RT).
• The resulting mixture was vortexed until the paste was fully dissolved and the microemulsion was allowed to sit for 20 min to allow air bubbles to coalesce.

## Graphical abstract

**Figure:**
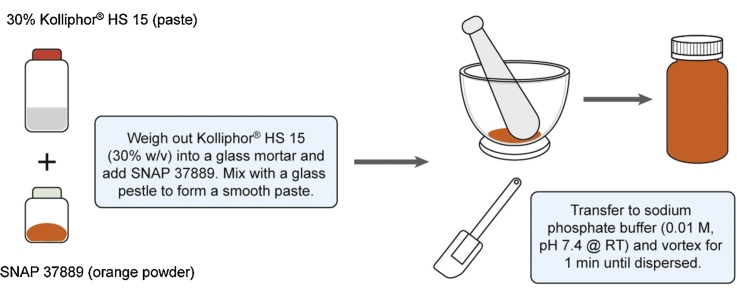

## Method details

### Troubleshooting

**Fig. 1:**
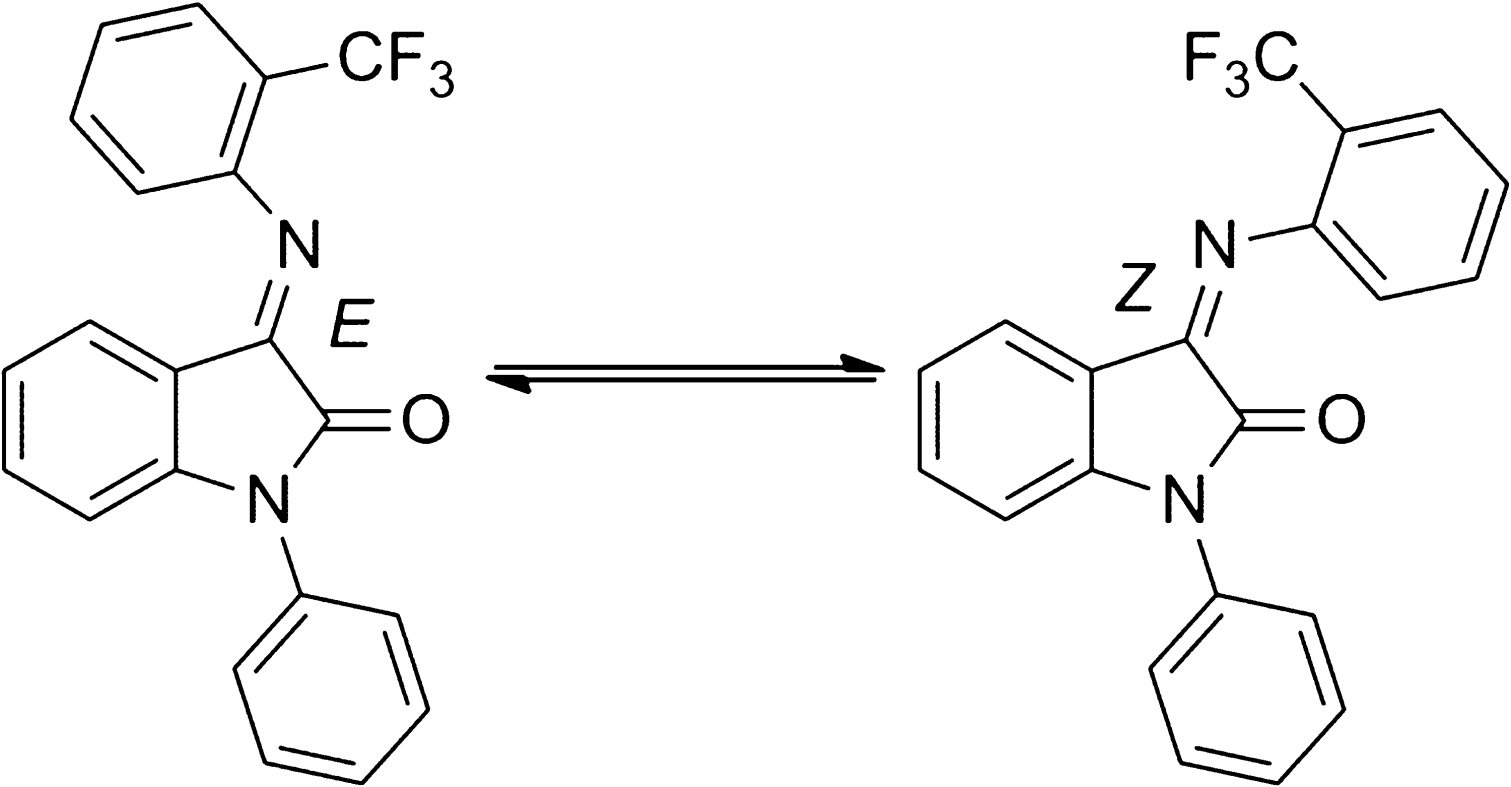
Interconversion of the *E*- and *Z*-isomers of SNAP 37889.

SNAP 37889 is a 3-arylimino-2-indolone derivative which when synthesised as previously described [1] forms an orange solid. 3-Arylimino-2-indolones exist as *E*- and *Z*-isomers (see Fig. 1) that interconvert rapidly in solution at room temperature. While it appears that the proportion of isomers is solvent dependent, it is unknown which isomer is the more active one. Although this drug has been useful in some assays, its poor water solubility (<1 μg/ml) limits its broader utility [2] and hinders further research. While SNAP 398299 is considered the more water-soluble analog of SNAP 37889, the chemical synthesis of this compound is much more challenging. A better way to formulate SNAP 37889 would enable its use at higher doses in a range of animal studies.

In previous studies [3,4] we utilized 5% DMSO with 1% hydroxypropylmethyl cellulose in saline, but the poor solubility of the drug in this vehicle remained a constant concern. We therefore investigated alternative formulations using this vehicle. This included heating the combined suspension to 100 °C, sonicating for short bursts (5–10 min) over 2 h and vortexing. These changes failed to improve the solubility of SNAP 37889. In addition, we tested substituting saline for water, as some salts can influence drug solubility, although again, this had little effect. We then established that the minimum percentage of DMSO needed to effect dissolution of SNAP 37889 at a dose of 10 mg/kg (which is lower than the effective dose used in our studies) was 60%. While there is no direct reference made to the amount of DMSO that can be used for injections in the Australian Code of Practice for the Care and Use of Animals for Scientific Purposes [5], it has been suggested that 0.5–5% is a suitable range [6]. Furthermore, while the medical use of DMSO has been widely documented, the toxicity of DMSO has been shown in several species, including humans (for review see [7]). For this reason, and since we were already using the maximum recommended dose of DMSO, a substitute for DMSO was therefore sought. We experimented with different types of modified complexing agents including 2-hydroxypropyl β-cyclodextrin and sulfobutylether-7-β-cyclodextrin which are safe to use in humans. Again, no improvement in the solubility of SNAP 37889 was obtained.

Next, we investigated vehicles for SNAP 37889 used in previous studies. First, 5% *N*,*N*-dimethylacetamide and 10% polyethylene glycol in water was examined [8]. SNAP 37889 precipitate was observed even at low concentrations. For example, 9 mg of SNAP 37889 in 250 μl *N*,*N*-dimethylacetamide and 500 μl polyethylene glycol resulted in precipitate when only 400 μl of water was added out of a final volume of 4, 250 μl. Therefore, this vehicle was not suitable for the higher doses of SNAP 37889 required for our study. Subsequently we examined 0.3% Tween 80 in Tris buffer, at pH 8.5 [9]. While this study did not report problems with solubility, no significant decrease in ethanol intake, anxiety or depression with SNAP 37889 was reported with this vehicle for doses of 10–30 mg/kg. We speculate that the oral route of administration used might have resulted in hydrolysis of the imine functional group during the drug's passage through the gastrointestinal system and degradation of the drug. Consequently the addition of acids or bases to help dissolve SNAP 37889 was avoided. It is worth noting that enteric coating could be applied to protect the imine functional group from hydrolysis in stomach acid for oral administration. Indeed, the success of enteric coating of the galanin-receptor 3 antagonist, HT-2157 (an alternative formulation of SNAP 37889), has been approved for use in human clinical trials for major depressive disorder [10].

**Fig. 2:**
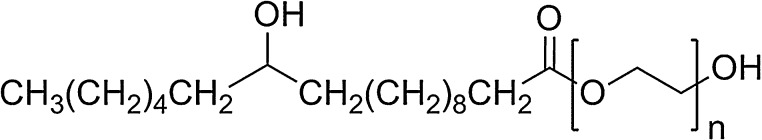
Structure of the non-ionic solubiliser Kolliphor^®^ HS 15. Synonyms include: Solutol HS^®^ 15, polyethylene glycol 15-hydroxystearate, macrogol 15-hydroxystearate, and polyoxyethylated 12-hydroxystearic acid.

**Fig. 3:**
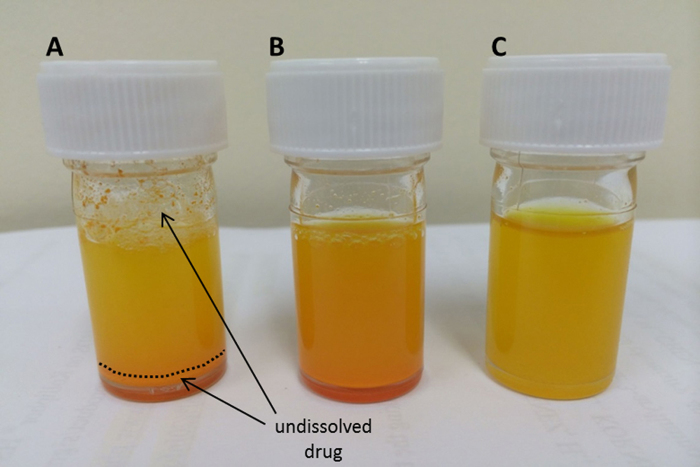
Comparison of vehicles for rat (injection volume 1 ml/kg) and mouse administration (injection volume 10 ml/kg) of SNAP 37889. (A) Rat: 150 mg of SNAP 37889 in 5 ml of 5% DMSO + 1% hydroxypropylmethyl cellulose in saline resulted in incomplete dissolution. (B) Rat: 150 mg of SNAP 37889 in 5 ml of 30% (w/v) Kolliphor^®^ HS 15 in 0.01 M sodium phosphate buffer (pH 7.4) formed a homogeneous microemulsion, which is comparable to (C) Mouse: 15 mg of SNAP 37889 in 5 ml of 30% (w/v) Kolliphor^®^ HS 15 in 0.01 M sodium phosphate buffer (pH 7.4).

**Fig. 4:**
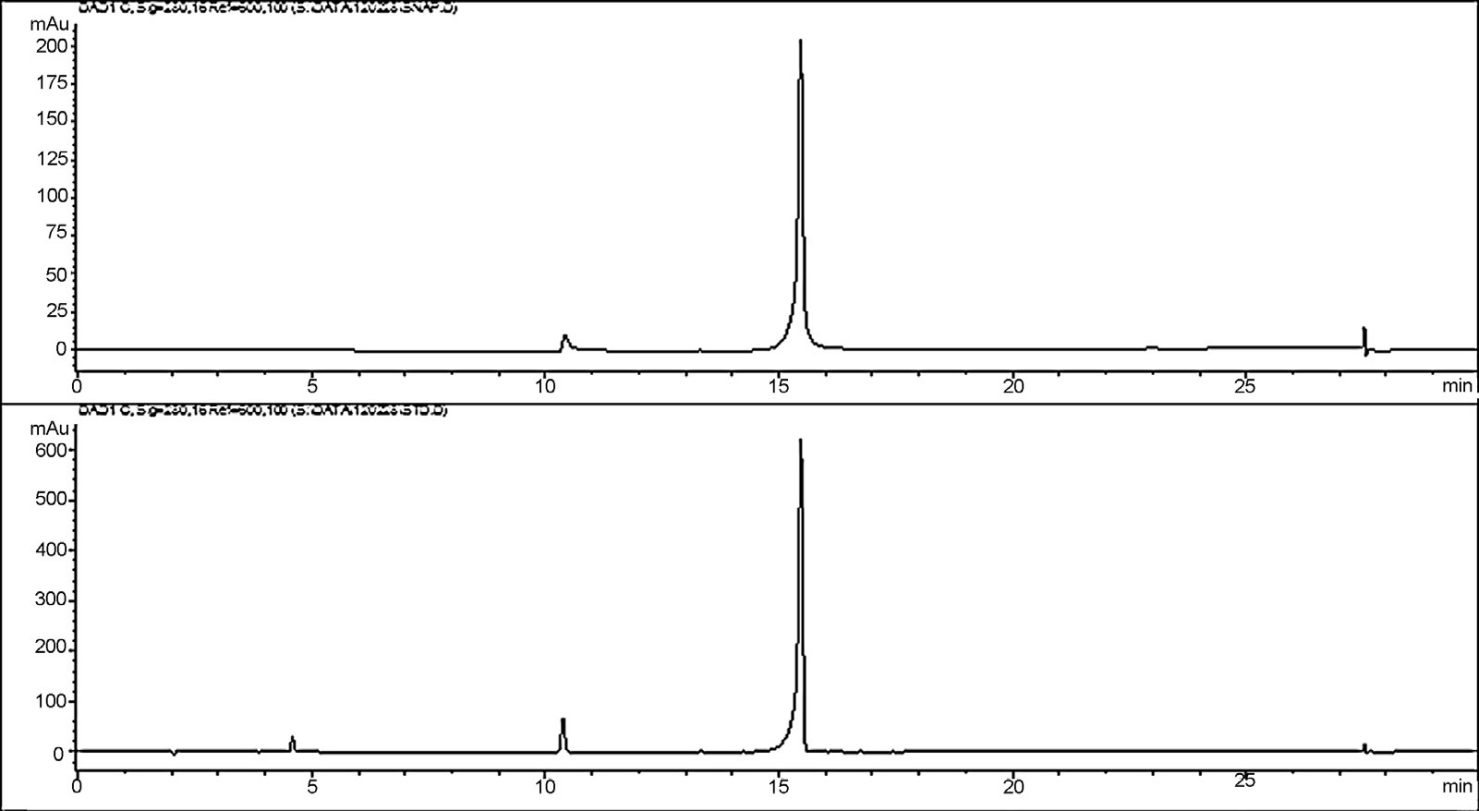
HPLC of SNAP 37889 in the Kolliphor^®^ HS 15-based vehicle (top panel) and control SNAP 37889 dissolved in saline (bottom panel). The peak at 10.3 min is an unidentified impurity.

Lastly, we examined the combination of 30% (w/v) Kolliphor^®^ HS 15 in 0.01 M sodium phosphate buffer (pH 7.4) as previously described [1] for subcutaneous injections in Sprague-Dawley rats. These workers heated a mixture of SNAP 37889 to 60 °C for 2 h prior to dosing. This particular vehicle is reported in the Supplementary Information file of that paper and we contacted the authors directly for more information. Kolliphor^®^ HS 15 is a non-ionic emulsifier (see Fig. 2), a white/colourless, odourless paste that is well tolerated and is used in the pharmaceutical industry in human and veterinary injection formulations. We found that simply vortexing a suspension of SNAP 37889 with a vehicle consisting of 30% (w/v) Kolliphor^®^ HS 15 and sodium phosphate buffer (0.01 M, pH 7.4) was sufficient to form a microemulsion which was eminently suitable for animal studies. Fig. 3 shows a comparison of the DMSO-based vehicle to this Kolliphor^®^ HS 15-based vehicle. The integrity of SNAP 37889 in the new vehicle was verified by HPLC (see Fig. 4), which showed essentially no degradation. Finally, mice (C57Bl6J, *n* = 50) were utilised to test this vehicle and they showed no signs of pain or adverse reactions after subcutaneous injections. This vehicle has subsequently been used in all further experiments in both rats and mice.

## Procedure

**(Values given to make a 10 ml emulsion for a 30 mg/kg dose of SNAP 37889 with an injection volume of 1 ml/kg.)**

1 Weigh out 3 g of Kolliphor^®^ HS 15 and place it in the bottom of a smooth glass mortar. Avoid use of a porous ceramic mortar and pestle as this leads to difficulties in the subsequent transfer. Kolliphor^®^ HS 15 is a thick paste that is conveniently transferred using a soft plastic spatula.
2 Weigh out 300 mg of SNAP 37889 and add on top of the paste.
3 Triturate components together thoroughly using the pestle until the visible particles of SNAP 37889 have been dissolved (approx. 2 min).
4 Using the spatula, transfer the drug paste and gradually add 6.7 ml of sodium phosphate buffer to make up to the final volume. Ensure that the buffer is at room temperature, as the paste will dissolve quicker if warm (body heat from holding the tube containing the solution in the palm of your hands helps). Vortex the final mixture until paste is dissolved (1 min). After vortexing, the solution will be aerated, as Kolliphor^®^ HS 15 is a detergent, so let the solution sit for 20 min or until most bubbles have coalesced. Note: if making the drug-free vehicle, mix the Kolliphor^®^ HS 15 paste alone in the mortar and pestle to soften before adding the sodium phosphate buffer.
5 Load syringes with appropriate volume ready for injection.

*Note*: Both SNAP 37889 and Kolliphor^®^ HS 15 are light sensitive and if stored in transparent vessels they should be wrapped in foil to exclude light.

## Additional information

### Background

Galanin is a peptide inhibitory transmitter widely expressed in the brain and gastrointestinal tract of mammals that signals through three G protein-coupled receptors: GAL1, GAL2 and GAL3 [11]. Two non-peptide compounds, SNAP 37889 and SNAP 398299, have suggested a role for GAL3 receptors in anxiety, depression and drug-seeking behaviour [8]. These compounds are substituted 3-arylimino-2-indolones that are poorly water soluble and difficult to inject parenterally. We have therefore developed an improved method for solubilising SNAP 37889 for injection into rats and mice to investigate its neuropharmacology in more detail.

The goal of drug treatment is to deliver a medication and sustain therapeutic levels at the site of action. When considering pharmacokinetics, drug absorption, metabolism and distribution will affect bioavailability of a drug. In the drug development industry, high-throughput screening methods have led to a growing number of lipophilic compounds whose therapeutic effectiveness is compromised by their low aqueous solubility [12]. For this reason, drugs are often prepared with the assistance of co-solvents or by altering the pH [13]. Furthermore a limitation of parenteral therapies is venous irritation and while its pathogenesis is not completely known, several contributing factors have been identified. These include properties of the final solution (for example pH, injection volume, tonicity, temperature, and concentration), the intrinsic nature of the drug, the injection procedure and the type of excipient (buffer or co-solvents) used in the solution (for review see [14]). Physical aspects such as the presence of particulates and precipitation of a drug can also cause venous irritation. If a drug is poorly water soluble, precipitation can occur when the drug is diluted in aqueous fluids, such as the blood [15]. Furthermore, crystals can form in the blood stream, changing bioavailability, as well as producing serious pain and phlebitis [16]. The use of oil-in-water parenteral emulsions can reduce or avoid many of these issues as their structure permits solubilisation of lipophilic agents in the oil phase, making them ideal vehicles for drug delivery [17].

The fundamental concept of lipid emulsions is to encapsulate a drug that has a strong affinity for lipids in globules that form tiny suspensions and via different methods dissolve these ‘loaded droplets’ at the desired sites of action [18]. These ‘micro’- and ‘nano’-emulsions provide a relatively inexpensive and simple alternative for drug delivery and are extensively used in the pharmaceutical industry due to their favourable biocompatibility, ability to control droplet size and decreased vein irritation [17,19,20]. The surfactant Kolliphor^®^ HS 15 has been successfully used to deliver lipophilic medications, venous irritant drugs and even genetic material [18,21,22] to the body via this way. Gan and colleagues [18] designed a microemulsion using Kolliphor^®^ HS 15, castor oil, glycerol and water to deliver a hydrophobic peptide cyclosporine A to the eye. Even 32 h after release of the microemulsions, cyclosporine A concentrations remained at therapeutic levels. Furthermore, tests to assess ocular irritation showed compatibility of the microemulsion [18]. Mao and colleagues [22] used an egg phospholipid and two co-emulsifiers, Poloxamer 188 and Kolliphor^®^ HS 15 to design a nanoemulsion to deliver lipophilic and venous irritant drugs. Mao and colleagues [22] found that their diallyl trisulfide nanoemulsion was an effective vehicle to deliver this compound and alleviated venous irritation allowing the treatment of systemic fungal and bacterial infections.

In conclusion, we have used the non-ionic surfactant Kolliphor^®^ HS 15, a promising excipient that can assist in delivering poorly water-soluble compounds by providing a stable microemulsion formation of SNAP 37889 in sodium phosphate buffer. This formulation provides an appropriate vehicle for administration into rodents for functional and mechanistic studies that overcome the problems with previously reported vehicles.

### Useful links

**•** General product information on Kolliphor^®^ HS 15 from Sigma–Aldrich website: http://www.sigmaaldrich.com/catalog/product/sigma/42966?lang=en&region=AU
• Link to PDF containing technical information on Solutol HS^®^ 15 (from the BASF chemical company): http://www.pharma-ingredients.basf.com/Statements/Technical%20Informations/EN/Pharma%20Solutions/03_030748e_Solutol%20HS%2015.pdf
